# A Friendly Alias

**Published:** 1887-07-30

**Authors:** 


### A Friendly Alias.

By W. J. E.

(*Concluded from p. 286, vol. ii.*)

Chapter IV.—Sorely Tried.

At first Kirsty affected not to see the tendency of affairs; but soon she was forced to recognise it. She gave Mr. Lely no encouragement. She could not help being affable, even friendly with him; but on any approach to a more serious relation, she became altogether deaf. Her friends gave gentle hints—

"So unreasonable, my dear! Not that I want to give advice. But when there's every compatibility—he's so kind, so nice, so clever. He's superior to Mr. Heathcote in every way; and as that engagement is at an end, why, there's no obstacle"—so said Mrs. MacPherson, with all the authority due to age and having known Kirsty since "she was in long clothes."

"Of course it's quite right to keep the men waiting a little," said a young married lady and former school-fellow of Kirsty. "It doesn't do to jump into their arms, so to speak. It makes them too conceited, and, goodness knows, they're conceited enough. But there is a limit."

And in similar strain spoke one well-meaning friend after another, till poor Kirsty's life became miserable. Her heart beat as true to Willie's as ever it did in those old, happy days.

In the first few weeks after her letter of renunciation, a hope of a speedy retractation on his part had filled her heart, but this hope had grown dimmer and dimmer, until she seemed to have nothing to buoy her up but her own undying love, and the memory of a long bygone happiness. She listened to Mr. Lely's addresses with a sad ear, and her manner towards him became gentle enough. She could not but appreciate the delicate tact with which he treated her. It almost seemed to her as if there was some undercurrent of accord between him and her, as if, in short, he knew the history of her one love-episode. She was troubled in her mind as to whether Willie still loved her, now he was so far away. She questioned herself again and again as to how she was to put an end to Mr. Lely's suit once for all. He didn't seem inclined to take a hinted "No," however strong, for an answer. She was nearly beside herself with the persecutions of her mother and her friends, who were totally incapable of seeing into her motives, since they were in possession of no facts but the one—viz., that she was free.

Was it to be wondered at if she became ill in body and mind? This fact Mr. Lely observed, and found it necessary to meditate over at a late hour for several evenings with the help of his favourite meerschaum. His meditations were generally in the open air, and it was one of these evenings as he stood on the "auld brig" over the river, near one end of it, and in the shadow cast by some thick birches, that he caught a glimpse of a white figure passing down the brae from Mrs. Seaton's house between the rhododendron bushes. He knew it must be some one passing down the garden from Gordon Brae. As the full light of the night, such as it was, caught some portion of her head and figure, he said to himself, half audibly, "What in the world! it's Kirsty!" Then he wondered what had brought her out at that uncanny hour. Had she wanted fresh air merely, she could have taken it better nearer the house. Still she came down nearer and nearer to the water. Whatever the thought was that flashed through Lely's brain, it caused him to clamber rapidly along the steep bank, whistling aloud, and suddenly confront Kirtsy, who stood as one dazed, with a "Lovely night, Miss Seaton!" as if he had almost expected to see her.

For a moment she shook from head to foot with the revulsion of thought. Then she burst into tears, at the same time clasping Lely's proffered arm, and then sobbed out:

"Oh! Mr. Lely! Do—do leave this place. I shall go mad if this goes on. I can never, never be your wife. Do not ask me why or how. I throw myself on your generosity. You have been kind to me, but every-one else has persecuted me till I am ill."

Lely was surprised at this outburst. She was evidently over-wrought by what she had passed through. He gently led her up the path as she spoke between her sobs, and, as she crossed the threshold, he held both her hands a moment, saying, "I will obey. In return, write to-morrow to Langley Heathcote, telling him that you are being urged to marry a rival of his; add whatever details you like except this, that I retire from the contest. Miss Seaton, I am your friend. Good-night!"

Kirsty was too much excited to sleep directly she reached her room. She therefore made another entry in her book:

"To-night horrible thoughts came into my head. I had been driven nearly mad by the entreaties, advices, persecutions of my friends, by my doubts and fears concerning Willie, by Mr. Lely's attentions. I could not go to bed. I went out, with what idea I don't know—am I becoming distraught?—but as I heard the splash of the river I thought again and again of those lines:

'Glad to death's mystery
Swift to be hurled—
Anywhere, anywhere
Out of the world,'

and I walked down the path towards the river, wondering all kinds of terrors. I almost believe I might have plunged in, if Mr. Lely had not sprung from somewhere and broken the spell. What could he think? He was kind, as usual. His last words still ring in my ears—'write to Langley Heathcote.' What does he know of Willie? There is some mystery about him, that's evident. I will write. How will this end?"

Next day Mr. Lely took an affectionate leave of Mrs. Seaton. Elsie seemed very loth to let him depart. Kirsty pressed his hand with a warm touch that thanked him,—he knew for what. It was noised that business had suddenly called him away, and that he would soon be back.

Chapter V.—How it all Ended.

Langley Heathcote had been in Havre a year. By dint of hard work and his own natural abilities he had mastered the details of the business of Clayton, Clayton, and Jarrow, so far as it related to the Havre agency.

He had gained the admiration of Mr. Jarvis, the agent, and, he believed, the firm's approval. In the busy French seaport he had acquired much useful commercial knowledge. But he had no grounds for expecting a career. There were many much older men, just as well qualified, drawing just the same salary. But of that he cared comparatively little. He had enough and to spare. There was, however, one care which always brooded over him, and that was Kirsty. Often he had tried to satisfy himself that she was all right, that she had by this time got over her first pangs, interested herself once more in such pursuits as make up a young lady's life. Her letters showed no other. Yet when he took that year old letter of hers out, as he did occasionally, his heart misgave him. Nevertheless he would not probe her feelings by letters. No! He told himself he had no right to do it now; and there was no prospect for him. *Cui bono?*

One morning he received at his lodgings two letters, one from the firm, in Clayton's hand, the other from Kirsty. He opened the firm's first. It was in Clayton's own handwriting, but formal, and signed with the firm's signature. It ran:—

"Dear Sir,—The firm are proposing to open a new agency at Bordeaux. From the accounts given us by Mr. Jarvis, and what we have seen from the business done in Havre since you took up your post there, we have every confidence that you can fill the new vacancy with credit to yourself and profit to the firm. With a view to receiving instructions in detail, please leave Havre at once, and come over, drawing on Mr. Jarvis for expenses. The salary we propose to offer at first is £300.—Yours faithfully,

"Clayton, Clayton & Jarrow."

Heathcote could scarcely credit it. He saw in it, of course, the hand of his friend Clayton. At the same time he knew Clayton would not have acted without the sanction of Mr. Jarrow, or without being convinced that Heathcote was fit and capable for the new responsibilities to be placed upon him. A thrill of pride at this early result of his conscientious efforts went through him.

Heathcote then turned to the other letter, and read:

"My dear Willie,—I am longing to see you. You cannot imagine what trouble and torture I have passed through these past six weeks. A Mr. Lely has been staying in the neighbourhood, and doing his best to supplant you. He has everything to recommend him. I have no arguments to use against him. Mother, sister, friends, are all on his side, urging me to marry him. Only my old love for you has supported me so far. I have been on the verge of desperation. I do not know what may happen if I have no one to help me. If you still love me, put aside all obstacles, and come to, once your own, "Kirsty."

The two letters, had they been planned, could not have been better judged, better timed. The added element of a rival in the field, indignation at anyone presuming to torture the mind of one who had been his Kirsty, the grand feeling of satisfaction at being able to approach her in his own right, were all so many sharp spurs to Langley Heathcote. Not long, it may be supposed, was he in parting from the genial Jarvis, and getting aboard the packet for Southampton. Then came the long railway journey, wherein the Flying Scotchman seemed, to his impatience, lazy. How he chafed at the delay at York, rejoiced when the furnaces of Darlington glared into the night as the train passed, and how eagerly he sprang to his feet as it crossed the High Level, and he could see up Tyne the red flames of Elswick Works! He had not forgotten to wire to Clayton, and, before the train stopped, he was shaking hands with that bronzed and hearty Northman. It was late when he reached the city, but at Clayton's house, after a "bit of supper," he found time before turning in to broach the subject nearest his heart after the Bordeaux scheme, which was, by mutual consent, postponed till the morning.

"The fact is, Cyril, I want to go North for a few days."

"Oh!" said Cyril, with a sly smile—and cleared his throat with unnecessary vigour.

"Don't be surprised, old fellow," Heathcote went on, "if I say I'm going to see Kirsty again."

"I'm only delighted," said Cyril. "But I say—I don't want to play gooseberry—but if there's any fishing in the neighbourhood, I'd like a few days."

"Come, by all means. My cup will overflow then. And I say, old man, if I hadn't known you to be a confirmed bachelor, there's a bewitching little sister."

"No! Is there? Further inducements. Really I must go. Let's see, we'll get through all the business with Jarrow, at least the preliminary arrangements, first thing to-morrow, and then start at two o'clock. We'll get to Strathhaven by night."

And the second morning after that, just at breakfast time, the two young men walked in at the garden gate, and nearly upset Miss Elsie, who, with a pair of steps, was trying to reach a rose high up on the side of the house.

"Mr. Lely! How are you?" was her first joyous exclamation: and then, with a cry of wonder, "Why? Willie! Mr. Heathcote, I mean!"

Heathcote looked at Clayton, then at Elsie, and then introduced his friend in a formal manner as "Mr. Cyril Clayton," which gentleman bowed with much gravity, and hoped she was well. Elsie was just on the point of asking an explanation, when Kirsty came suddenly out to call Elsie in to breakfast. Heathcote was half-hidden behind the corner of the house. She blushed, and faltered, "How do you do, Mr. Lely?" Clayton caught Heathcote's arm, and led him a step forward, saying as he did so:

"Miss Seaton—a present from Havre! Come, Miss Elsie, have you any breakfast for me?" and he linked his arm in hers and left the two reunited lovers in the freshness of the morning to greet one another in their own fashion.

How Mr. Cyril Clayton's powers of fascinating were exerted with Mrs. Seaton on his friend's behalf, at the same time that he pleaded an utter want of success as his own excuse for not prosecuting his suit, need not be told. He had a harder task in explaining the whole history of the ruse and its entire innocence to his friend Heathcote, who was much inclined to be jealous.

For the fortnight they were there Mr. Cyril Clayton was not a success as a fisherman. Not that he was idle, but, as he said, the fish didn't come his way. They showed a want of taste. If they did, Elsie made up for it. For where Elsie was, Cyril Clayton was to be found somewhere near,—or shall I put it *vice versâ?* And it took a terrible amount of explaining on the part of Mr. Clayton that, though Mr. Lely might pay attentions to Miss Kirsty in order to bring about certain results, Mr. Clayton was in no wise compromised, and was, in fact, free to offer a hitherto unaffected bachelor heart to Miss Kirsty's younger sister. But at last even Elsie agreed that she understood the matter. And "mammas" in Newcastle were very "surprised" to hear that Mr. Clayton was shortly going to marry a young lady from Scotland, of "quite an old family," they believed, and that it was "wholly a love-affair,"—as if marriage should ever be anything else.

